# Sustainable water reuse in food production: risks of extended-spectrum β-lactamase (ESBL)-producing *Escherichia coli* and antimicrobial resistance gene release from tertiary-treated reclaimed water

**DOI:** 10.3389/fmicb.2025.1591202

**Published:** 2025-06-19

**Authors:** Pilar Truchado, Márcia Oliveira, Rebeca Cordero-García, Manuel Abellán Soler, Amador Rancaño, Francisca García, Avelino Álvarez-Ordóñez, Ana Allende

**Affiliations:** ^1^Research Group on Microbiology and Quality of Fruit and Vegetables, Food Science and Technology Department, CEBAS-CSIC, Murcia, Spain; ^2^Department of Food Hygiene and Technology and Institute of Food Science and Technology, Universidad de León, León, Spain; ^3^Entidad Regional de Saneamiento y Depuración de Murcia (ESAMUR), Murcia, Spain; ^4^Acciona Agua, S.A.U., Madrid, Spain

**Keywords:** wastewater treatment, sustainable agriculture, ESBL-*E. coli*, antimicrobial resistance (AMR), irrigation water

## Abstract

Wastewater reuse for agricultural irrigation is increasingly essential, but it carries potential public health risks due to the dissemination of antimicrobial resistance (AMR). This study evaluates the effectiveness of four tertiary wastewater treatment technologies—peracetic acid (PAA), PAA combined with low-intensity ultraviolet-C (PAA/UV Low), high-intensity UV-C (UV High), and ultrafiltration (UF)—in reducing extended-spectrum β-lactamase-producing *Escherichia coli* (ESBL-*E. coli*) and antimicrobial resistance genes (ARGs) in reclaimed water used for irrigation. The relative abundance of the genes, normalized to the 16S RNA gene present in the water samples, was then estimated to assess whether there is an amplification of these genes during the reuse process in the wastewater treatment plant (WWTP). The results indicate that while all treatments significantly reduced ESBL-*E. coli* (≥3 logs cfu/100 mL) and ARGs (≥ 1.5 logs gc/100 mL), complete elimination was not achieved in any WWTP. Among the treatments, UF demonstrated the highest removal efficiency (≈4 log gc ARG/100 mL), against ARGs, followed by UV High (≈3 log gc ARG/100 mL), whereas PAA and PAA/UV Low were less effective (≈2 log gc ARG/100 mL). The study also found that while absolute ARG levels were reduced, their relative abundance remained stable or showed minimal decline, suggesting a persistent environmental reservoir of resistance genes. Among the ARGs analyzed, the most frequently detected were associated with tetracyclines (*tetW, tetA*), quinolones (*qnrB, qnrS*), and sulfonamides (*sul1, sul2*), highlighting potential public health concerns. Moreover, multidrug-resistant (MDR) ESBL-*E. coli* isolates were present across all WWTPs, exhibiting resistance to β-lactams, quinolones, tetracyclines, and sulfonamides. Nevertheless, notably low levels of resistance to last-resort antibiotics (tigecycline, colistin, and meropenem) were observed. These findings underscore the critical role of tertiary treatments in mitigating antimicrobial resistance (AMR) risks in water reuse systems. However, the persistence of ARGs in effluents suggests that current WWTP processes require further optimization.

## Highlights

- Multidrug-resistant ESBL-*E. coli* persisted in WWTP effluents.
- ARGs were reduced but not completely eliminated in treated wastewater.
- Ultrafiltration (UF) was the most effective in reducing ESBL-*E. coli* and ARGs.
- High-dose UV was more effective than PAA and PAA/UV Low for ARG removal.
- Detection of ARBs and ARGs emphasizes the need for improved water reuse strategies.

## 1 Introduction

Antimicrobial resistance (AMR) is a significant global public health concern, as bacteria carrying antimicrobial resistance genes (ARGs) and deployment of antibiotic residues are the main contributing agent causing thousands of deaths each month (GBD 2021 Antimicrobial Resistance Collaborators, 2024). The WHO considers this threat a priority and has endorsed a global action plan on AMR, which includes the development of a global public health research agenda to address major knowledge gaps [(World Health Organization (WHO), 2018)]. One contributing factor to this crisis is the excessive and inappropriate use of antibiotics in medical, livestock, and agricultural contexts (McConnell et al., 2018). This has led to the continuous deployment of antibiotic residues into the environment, facilitating the emergence of antimicrobial resistant bacteria (ARB) and ARGs. Europe, and particularly, the European Commission places significant importance on addressing AMR due to its critical impact on public health, healthcare systems, and economies.

The support that the European Commission gives to the One Health approach recognizes that human health, animal health, and the environment are all interconnected. AMR is a prime example of this interdependence, as resistant microbes and resistance genes can be transmitted between humans, animals, crops, and the environment. This recognition is embedded in several key policy and legislative instruments, including Regulation (EU) 2019/6 on veterinary medicinal products, which introduces stricter measures to reduce the use of antimicrobials in animals, and Regulation (EU) 2019/4 on medicated feed. Moreover, the European Commission's 2019 Communication on the Strategic Approach to Pharmaceuticals in the Environment highlights the environmental dimension of AMR, promoting actions to address the release of pharmaceuticals, including antibiotics, throughout their life cycle. These efforts are further supported by the European One Health Action Plan against AMR (2017) and integrated into the broader Farm to Fork Strategy and the Zero Pollution Action Plan, which aim to reduce pollution from antimicrobial substances in water, soil, and food systems (European Commission, 2017). Wastewater treatment plants (WWTPs) receive wastewater from various sources, including municipalities, hospitals, and industries, and have been underscored as potential hotspots for the transmission of ARGs and, consequently, ARB (Hazra et al., 2024; Li et al., 2022). The main hypothesis is that wastewater contains antibiotics, high bacterial loads, and nutrient-rich substances, creating ideal conditions for the selection and spread of ARGs and ARBs, which confer resistance and survival advantages to microbial communities (Buriánková et al., 2021; Haberecht et al., 2019; Oliveira et al., 2018). While WWTPs design allows a successful removal of dissolved nutrients and solids, including bacteria, there is currently no specific technology in place for the targeted reduction of ARGs (Gao et al., 2022; Rizzo et al., 2013). Several studies have focused on evaluating the presence of ARB and ARGs in the influent and effluent of various WWTPs (Macrì et al., 2024; Sanz et al., 2024). Overall, most of these studies have reported a decrease in absolute concentrations after treatment. Generally, wastewater treatment reduces the overall abundance of ARBs and ARGs by ~2–3 logs, thereby lowering the risk of ARG dissemination into the environment (Ben et al., 2017; Hultman et al., 2018; Rodriguez-Mozaz et al., 2014). However, some studies have suggested that WWTPs may contribute to the proliferation of ARGs in the environment (Kumar et al., 2020; Stachurova et al., 2021; Wang et al., 2020).

The current European regulatory framework includes regulations on the use of antimicrobials in human and veterinary medicine, as well as measures to prevent the spread of resistant bacteria in healthcare settings and the environment, including water reuse systems [European Commission, 2020a; World Health Organization (WHO), 2018]. However, there is growing awareness of its connection to climate change and how climate change can influence AMR in agriculture (van Bavel et al., 2024). The EFSA Scientific Opinion [(EFSA (European Food Safety Authority, Panel on Biological Hazards), 2021)] further underscores the major role of the environment in the emergence and spread of AMR through food chains, identifying key transmission routes such as fecal-origin fertilizers, irrigation water, and animal feed. The Opinion also highlights the presence of critical ARB and ARGs [e.g., *bla*_CTX − M_, *mcr, van*(A), *qnr*] across plant-based and animal production systems, emphasizing the importance of mitigation strategies across sectors.

In Mediterranean countries, which are severely affected by water scarcity, Europe is promoting the use of reclaimed water for irrigation as part of a sustainable water management strategy (Echaide et al., 2021; European Commission, 2020b). Nonetheless, ensuring the microbiological quality of reclaimed water is crucial by monitoring not only pathogenic bacteria but also ARB and ARGs, as they could potentially be transferred to crops through irrigation (Macrì et al., 2024; Rodriguez-Mozaz et al., 2014; Sanz et al., 2024). However, the effectiveness of different water treatment technologies in reducing or eliminating ARBs and ARGs in WWTPs remains uncertain due to insufficient data (Ben et al., 2017; Osińska et al., 2020; Wang et al., 2020).

This study aims to investigate and compare various water treatments implemented in four distinct WWTPs for the reclamation of urban wastewater used in agriculture. The methods include peracetic acid (PAA), PAA combined with low-intensity ultraviolet-C (UV-C; PAA/UV Low), high-intensity UV-C (UV High), and ultrafiltration (UF). The research will evaluate the efficacy of these water treatments in reducing ARB carrying ARGs, with a particular focus on ESBL-producing *Escherichia coli* and 10 different ARGs. The assessment will be conducted using quantitative real-time PCR (qPCR).

## 2 Material and methods

### 2.1 Urban wastewater treatment plants

Four WWTPs located in the Region of Murcia, Spain, were selected for this study, each employing different tertiary treatment technologies. The primary and secondary treatment processes were previously described by Oliveira et al. (2023). Briefly, primary treatment typically includes aeration, solids and suspended solids separation, grit removal/degreasing, and a primary clarifier of varying size. Secondary treatment involves an aerobic or anaerobic biological process in a secondary clarifier, with coagulation/flocculation and supplementary lamella clarification. For tertiary treatment, the four WWTPs utilize different water treatment technologies: peracetic acid (PAA), PAA combined with low-intensity ultraviolet-C (UV-C; PAA/UV Low), high-intensity UV-C (UV High), and ultrafiltration (UF). The doses of PAA and UV are summarized in Table 1. Each WWTP receives both domestic and agricultural wastewater. Additionally, the WWTP using PAA also treats a significant amount of pre-treated hospital wastewater.

**Table 1:** Water treatments used in the WWTPs included in this study.

**WWTPs**	**Dates**	**Doses**	**Treatments**
1	June 2021– November 2022	5 mg/L	PAA
2	June 2021– November 2022	0.7–1 mg/L 20–40 mJ/cm^2^	PAA/UV low intensity (5–10 mW/cm^2^; PAA/UV Low)
3	June 2021– September 2022	100–1,020 mJ/cm^2^	UV high intensity (20–72 mW/cm^2^; UV High)
4	December 2021– November 2022		Ultrafiltration (UF)

### 2.2 Sampling

From June 2021 to December 2022, monthly influent and effluent samples were collected from each WWTP. However, UF samples were only collected from December 2021 to December 2022. A total of 132 samples were included in this study: 18 influent and 18 effluent samples were collected from each WWTPs, except for the UF system, from which 12 influent and 12 effluent samples were taken. One liter of each sample was collected in sterile polypropylene plastic bottles (Labbox Labware S.L., Barcelona, Spain), stored under refrigerated conditions, transported to the laboratory within 2 h, and kept at 4°C until analysis.

### 2.3 Presence of ESBL-producing *E. coli* in wastewater samples

The levels of extended-spectrum β-lactamase (ESBL)-producing *E. coli* (cfu/100 mL) in each influent water sample were determined by plating. Serial 10-fold dilutions were prepared in buffered peptone water (BPW, 2 g/L; Oxoid) and then spread-plated onto CHROMagar ESBL (CHROMagar, Paris, France). For effluent water samples, aliquots of 1, 10, and 100 mL were filtered through sterile cellulose nitrate filters (0.45 μm, Sartorius, Madrid, Spain) using a vacuum filtration system (Sartorius). Dark pink to reddish colonies were counted after 24 h of incubation at 37°C. All analyses were performed in duplicate, and results were expressed as log cfu/100 mL.

### 2.4 Antibiotic susceptibility testing

A total of 366 *E. coli* isolates producing ESBL were selected for antibiotic susceptibility testing (AST). The isolates were obtained from both influent and effluent samples across the four wastewater treatment processes (PAA, PAA/UV Low, UV High, and UF). The number of isolates tested per sample was proportionally selected based on the initial recovery rates of presumptive ESBL-producing *E. coli*, ensuring representative coverage of all water matrices. Susceptibility to a panel of antibiotics was assessed using Sensititre EUVSEC3 plates (Thermo Scientific, TREK Diagnostic Systems Ltd., East Grinstead, UK) and the broth microdilution method, following the manufacturer's instructions, as previously described by Oliveira et al. (2023). Briefly, isolates were cultured in BHI broth at 37°C for 24 h. The resulting bacterial suspension was adjusted to a 0.5 McFarland turbidity standard and transferred to Mueller-Hinton broth. Subsequently, 50 μL of the suspension were dispensed into each well of the AST plate using the Sensititre AIM Automated Inoculation Delivery System (Thermo Scientific, TREK Diagnostic Systems Ltd., East Grinstead, UK). After 24 h of incubation at 37°C, growth was visually assessed to determine the minimum inhibitory concentration (MIC) for each antibiotic. Interpretation of susceptibility or resistance was based on the epidemiological cut-off values (ECOFF) provided by the European Committee on Antimicrobial Susceptibility Testing (EUCAST). The antibiotics tested included sulfamethoxazole, trimethoprim, ciprofloxacin, tetracycline, meropenem, azithromycin, nalidixic acid, cefotaxime, chloramphenicol, tigecycline, ceftazidime, colistin, ampicillin, and gentamicin.

### 2.5 Wastewater DNA extraction

Ten milliliters of influent samples were concentrated by centrifugation at 3,000 × g for 10 min. The supernatant was removed, and the pellet was resuspended in 1 mL of phosphate-buffered saline (PBS, Sigma-Aldrich, LS, USA). The resuspended pellet was then centrifuged at 9,000 × g for 10 min at 4°C, and the supernatant was discarded. For effluent samples, 100 mL of water was vacuum-filtered through sterile cellulose nitrate filters (0.45 μm). The filters were placed in 50 mL Falcon tubes containing 20 mL of PBS supplemented with Tween 80 (1 mL/L; Sigma-Aldrich). The tubes were vortexed for 7 min, after which the filters were removed. The sample was then centrifuged at 3,000 × g for 10 min, the supernatant was discarded, and the pellet was resuspended in 1 mL of PBS. The resuspended pellet was further concentrated by centrifugation at 9,000 × g for 10 min at 4°C. Both influent and effluent pellets were stored at −20°C until genomic DNA extraction. Genomic DNA extraction from wastewater concentrates was performed using the Maxwell^®^ RSC Instrument (Promega) and the Maxwell RSC Pure Food GMO and Authentication Kit (Promega), following the Maxwell RSC Viral Total Nucleic Acid running program. DNA concentration and purity were determined using an Implen NanoPhotometer N60/50 (Implen, Munich, Germany). The DNA samples were then stored at −20°C.

### 2.6 Antimicrobial resistance genes qPCR assays

Quantitative PCR (qPCR) was used to quantify ARGs encoding resistance to beta-lactams (*bla*_CTX − M_ and *bla*_TEM_), chloramphenicol (*cmlA* and *catI*), quinolones (*qnrB* and *qnrS*), sulfonamides (*sul1* and *sul2*), and tetracyclines (*tetA* and *tetW*). Additionally, the 16S rRNA gene was analyzed to quantify total bacterial populations and normalize ARG abundance in the collected samples. All qPCR assays were performed using a QuantStudio 5 system (Applied Biosystems, USA) in 96-well plates with KAPA SYBR FAST and KAPA PROBE FAST Universal qPCR Master Mix kits (KapaBiosystems, Massachusetts, USA). The selection of primers and probes used to quantify ARGs as well as cycling parameters, are detailed in Table 2. Each qPCR analysis was conducted in triplicate wells containing both undiluted and diluted DNA samples. Wastewater samples were diluted 50-fold, while reclaimed water samples were diluted by a factor of 5. Each qPCR assay included three negative control wells (nuclease-free water). When SYBR Green was used for detection, a melting curve analysis was performed after each assay to confirm that the fluorescence signal originated from a specific PCR product. The standard curve for each ARG was established following the method described by Truchado et al. (2016). The limit of detection (LOD) and limit of quantification (LOQ) were determined for each target gene through a series of dilutions, with 4–10 replicates per dilution. ARG quantification results were expressed as absolute and relative abundance. Absolute abundance was reported as the number of copies per 100 mL of sample, while relative abundance was calculated by normalizing the absolute copy number of each gene to 16S rRNA gene copy numbers (i.e., ARG copies/16S rRNA copies). All values were log-transformed.

**Table 2:** Selected primers and probes used to quantify ARGs and the cycling parameters us in the PCR.

**Antibiotic group**	**Gene**	**Primer and probes (5**^**′**^→**3**^**′**^**)**		**Cycling parameters**	**References**
*β-lactams*	*bla*_CTX − M−G1_	FW	ACCAACGATATCGCGGTGAT	95°C for 10 min; 45 cycles of (95°C for 30 s, 60°C for 1 min)	Colomer-Lluch et al., 2011
		RV	ACATCGCGACGGCTTTCT		
		Probe	FAM-TCGTGCGCCGCTG-MGBNFQ		
	*bla*_TEM_	FW	CATTTCCGTGTCGCCCTTATTC	50°C for 2 min; 95°C for 3 min; 40 cycles of (95°C for 5 s, 60°C for 30 s, 72°C for 1 min)	Dallenne et al., 2010
		RV	CGTTCATCCATAGTTGCCTGAC		
sulfamethoxazole	*sul1*	FW	CCGTTGGCCTTCCTGTAAAG	95°C for 10 min; 45 cycles of (95°C for 30 s, 58°Cr 1 min)	Ginn et al., 2021
		RV	TTGCCGATCGCGTGAAT		
		Probe	FAM-CAGCGAGCCTTGCGGCGG-BHQ1		
	*sul2*	FW	GCGCTCAAGGCAGATGGCATT	50°C for 2 min; 95°C for 3 min; 40 cycles of (95°C for 5 s, 69°C for 30 s, 72°C for 1 min)	Aarestrup et al., 2003
		RV	GCGTTTGATACCGGCACCCGT		
*chloramphenicol*	*catI*	FW	GGTGATATGGGATAGTGTT	50°C for 2 min; 95°C for 3 min; 40 cycles of (95°C for 5 s, 55°C for 30 s, 72°C for 1 min)	Ng et al., 2014
		RV	CCATCACATACTGCATGATG		
	*cmlA*	FW	GCCAGCAGTGCCGTTTAT	50°C for 2 min; 95°C for 3 min; 40 cycles of (95°C for 5 s, 60°C for 30 s, 72°C for 1 min)	Liu et al., 2020
		RV	GGCCACCTCCCAGTAGAA		
*quinolones*	*qnrB*	FW	GATCGTGAAAGCCAGAAAGG	50°C for 2 min; 95°C for 3 min; 40 cycles of (95°C for 5 s, 50°C for 30 s, 72°C for 1 min)	Cummings et al., 2011
		RV	ATGAGCAACGATGCCTGGTA		
	*qnrS*	FW	GTATAGAGTTCCGTGCGTGTGA	50°C for 2 min; 95°C for 3 min; 40 cycles of (95°C for 5 s, 55°C for 30 s, 72°C for 1 min)	Tan et al., 2018
		RV	GGTTCGTTCCTATCCAGCGATT		
*tetracyclines*	*tetA*	FW	CCGCGCTTTGGGTCATT	95°C for 10 min; 45 cycles of (95°C for 30 s, 56°C for 1 min)	Ginn et al., 2021
		RV	TGGTCGCGTCCCAGTGA		
		Probe	FAM-TCGGCGAGGATCG-BHQ		
	*tetW*	FW	AAAACTTATTATATTATAGTG	50°C for 2 min; 95°C for 3 min; 40 cycles of (95°C for 5 s, 60°C for 30 s, 72°C for 1 min)	Aminov et al., 2001
		RV	TGGAGTATCAATAATATTCAC		
*16S RNA*	*16S*	F1048	GTGSTGCAYGGYTGTCGTCA	50°C for 2 min; 95°C for 3 min; 35 cycles of (95°C for 5 s, 60°C for 30 s, 72°C for 1 min)	Maeda et al., 2003
		R1194	ACGTCRTCCMCACCTTCCTC		

### 2.7 Statistical analysis

Treatment efficacy was calculated as follows: Log10 influent (cfu or gene copies/100 mL) – Log10 effluent (cfu or gene copies/100 mL). Graphical representations were created using R software (R Core Team, 2021) and SigmaPlot 14 (Systat Software, Inc., Addilink Software Scientific, S.L., Barcelona, Spain).

The Shapiro-Wilk test was performed to assess the normality of the data (*P* > 0.05). For normally distributed data, an ANOVA test was conducted, and when significant differences were detected, Tukey's HSD (Honestly Significant Difference) test was applied. The Student's *t*-test was used to compare the relative abundance of ARGs between influent and effluent samples.

## 3 Results and discussion

### 3.1 Effectiveness of reclamation treatments in removing ESBL-producing *E. coli* from WWTPs

**Figure 1:**
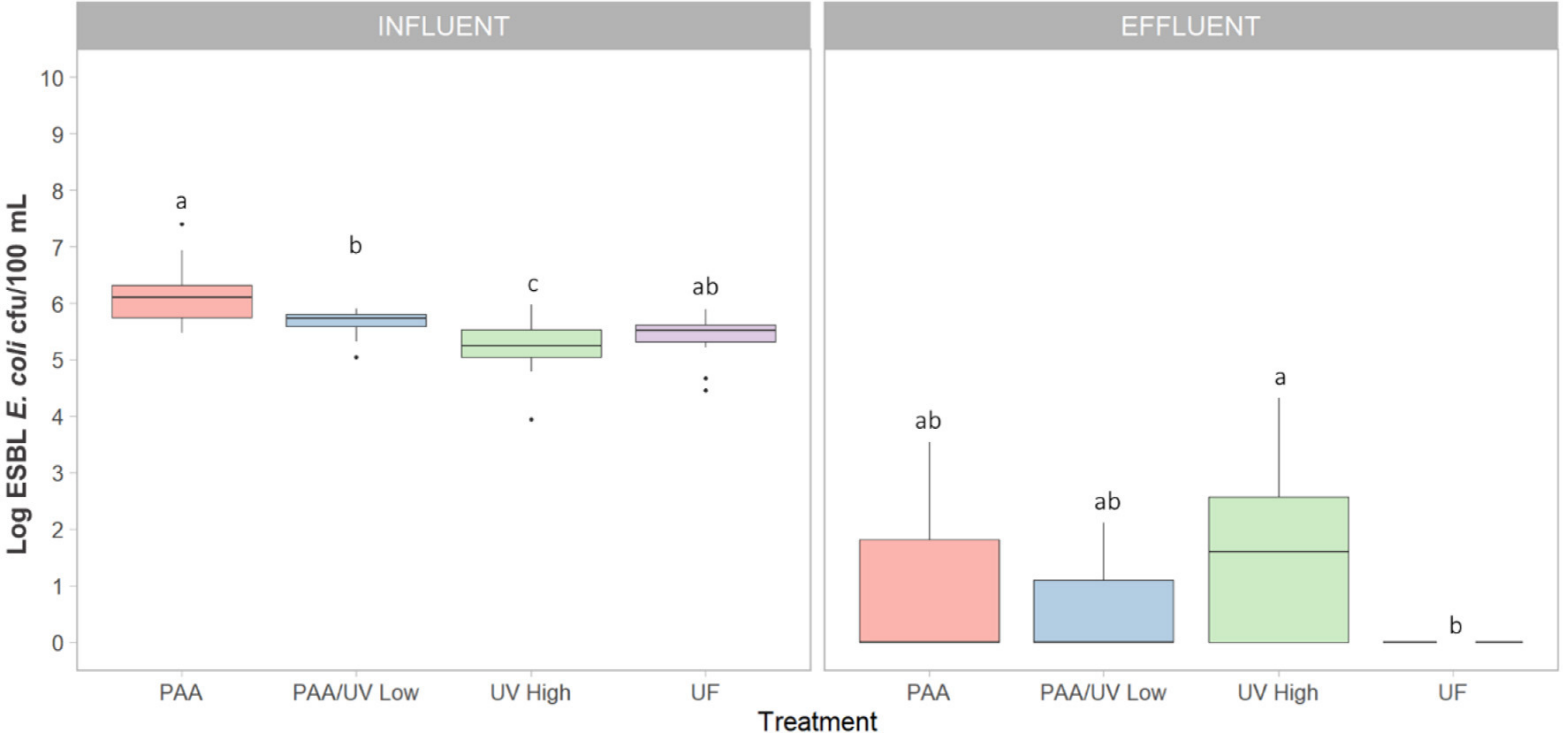
Level of ESBL-producing *E. coli* detected in the four WWTPs using different treatments. Box plots shows the median with the 25th and 75th percentile values of reduction. Box plots labeled with different letters indicate significant differences among treatments at *P* < 0.05.

ESBL-producing *E. coli* was detected in all influent samples from the four WWTPs (Figure 1). The median counts in influent samples were 6.1 ± 0.0, 5.7 ± 0.1, 5.2 ± 0.0, and 5.4 ± 0.0 log cfu/100 mL for WWTPs PAA, PAA/UV Low, UV High, and UF, respectively (Figure 1). These concentrations are in line with those reported in previous studies on untreated urban wastewater (Haberecht et al., 2019; Schmiege et al., 2021; Xie et al., 2023), indicating that our findings are consistent with values observed in similar settings.

On the other hand, differences were observed among wastewater samples, which may be influenced by various factors such as antibiotic usage patterns, population density, industrial and agricultural activities, proximity to hospitals, waste management practices, and mobility patterns. While we do not have direct evidence to determine the specific contribution of each factor, these represent plausible hypotheses that warrant further investigation. Notably, the highest levels of ESBL-producing *E. coli* were detected in the PAA WWTP, which is located near a hospital. This raises the possibility that hospital effluents may contribute to an increased presence of ESBL-producing *E. coli* in municipal WWTPs, as previously suggested by Lépesová et al. (2020).

After treatment, the ESBL-producing *E. coli* levels in effluent samples showed a notable reduction (Figure 1), with significant differences observed among treatments. In most WWTPs (PAA, PAA/UV Low, and UF), average counts were below 1.0 log cfu/100 mL, with several samples falling below the detection limit (0 log cfu/100 mL or 1 cfu/100 mL), particularly in the case of UF treatment. UF's effectiveness can be attributed to its ability to retain bacteria, given that their size exceeds the pore size of UF membranes (Michael et al., 2022). However, UV High treatment showed median counts exceeding 1.4 log cfu/100 mL (Figure 1), which is concerning as it sometimes surpasses the limits set by Regulation (EU) 2020/741 for water quality intended for edible agricultural crops (10 cfu/100 mL for *E. coli*). It is important to note that this regulation focuses on microbial indicators such as *E. coli* but does not include controls for ARB or ARGs. The observed high counts in UV High-treated effluent may be due to incomplete treatment during the early phase of sampling (June 2021–March 2022), before the full implementation of UV and PAA treatments. Raven et al. (2019) also reported ESBL-producing *E. coli* levels ranging from 2 to 21 cfu/mL in reclaimed water after UV treatment.

The detection of samples below the limit of detection at several sampling points demonstrates the potential effectiveness of certain water treatments in significantly reducing ESBL-*E. coli* loads. This finding is consistent with previous studies that reported substantial reductions in *E. coli* concentrations by up to 6 log units, through biological and tertiary treatments, including chlorination and UV-based systems (Xie et al., 2023). However, the persistence of ESBL-*E. coli* in final effluents, even at low levels, remains a concern. Similarly, Bréchet et al. (2014) found that urban wastewater contained high concentrations of ESBL-*E. coli*, with WWTP treatment reducing the load by ~95%. The authors also highlighted the genotypic overlap between clinical and environmental isolates, suggesting potential dissemination from hospitals into the environment. In the Spanish context, Oliveira et al. (2023) observed that although tertiary treatments such as UV and PAA-UV combinations resulted in significant reductions, ESBL-*E. coli* was still detected in several effluent samples, particularly where treatment was incomplete or inconsistent. Notably, the resistance profiles of isolates remained largely unchanged before and after treatment, indicating that current WWTP processes may reduce bacterial counts without fully eliminating resistance determinants. However, high levels of ESBL-producing *E. coli* have also been reported in both influent (4.5 ± 2.9 cfu/mL) and effluent (61–49 cfu/mL) samples treated with chlorine in a WWTP in Japan (Azuma et al., 2022). Similarly, Nzima et al. (2020) reported influent counts ranging from 4.1 to 4.2 log cfu/mL and effluent counts between 2.5 and 3.3 log cfu/mL. These findings collectively underscore the importance of ongoing surveillance and the optimization of advanced treatment technologies to prevent the environmental dissemination of ARB and ARGs.

### 3.2 Antibiotic resistance profile of ESBL-producing *E*. coli isolates

**Figure 2:**
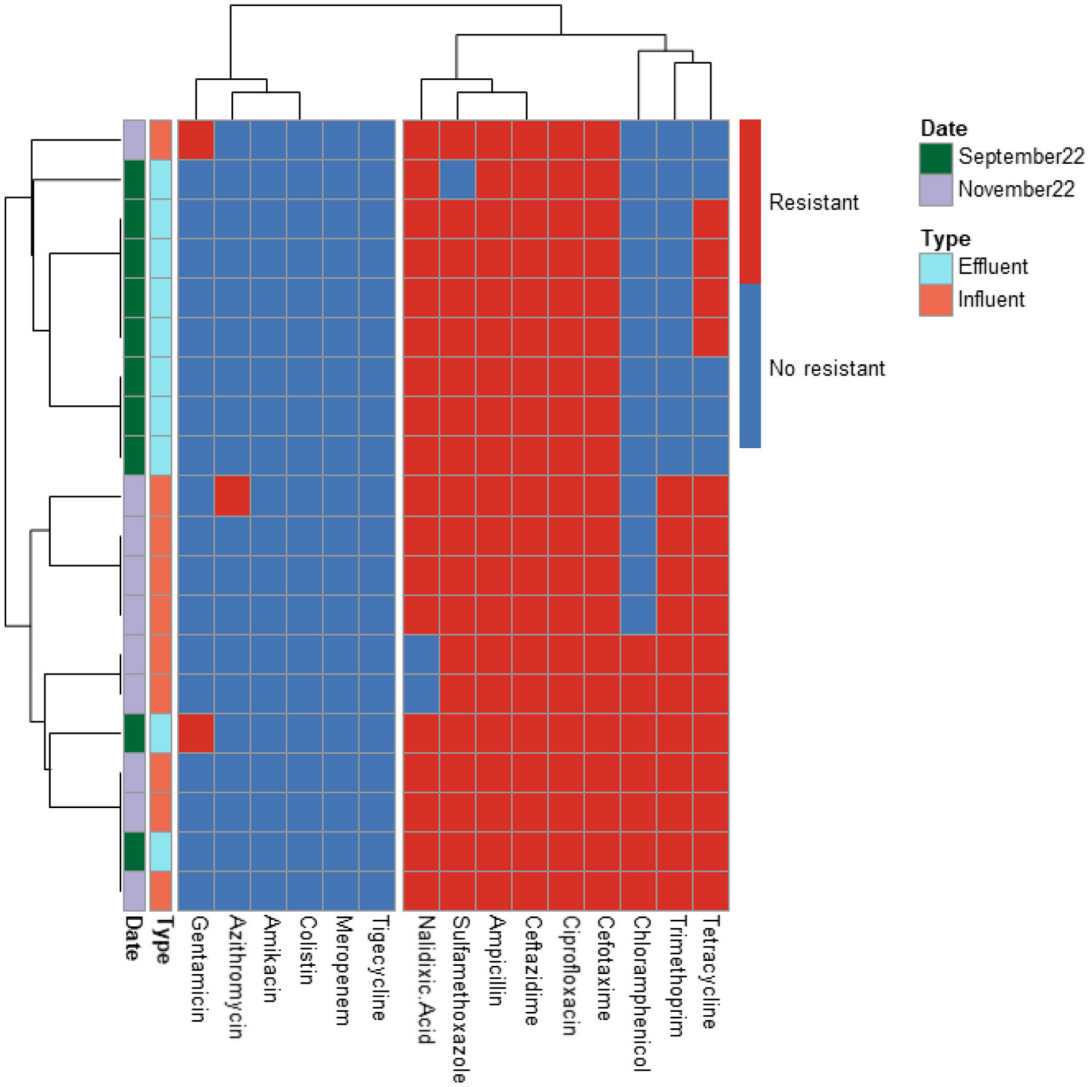
Heatmap displaying the antibiotic resistance profile of ESBL-producing E. coli isolates retrieved from PAA WWTP. The isolates (*n* = 20) were classified as antibiotic resistant (in red) or susceptible (in blue) based on ECOFF values from EUCAST, which served as the threshold.

**Figure 3:**
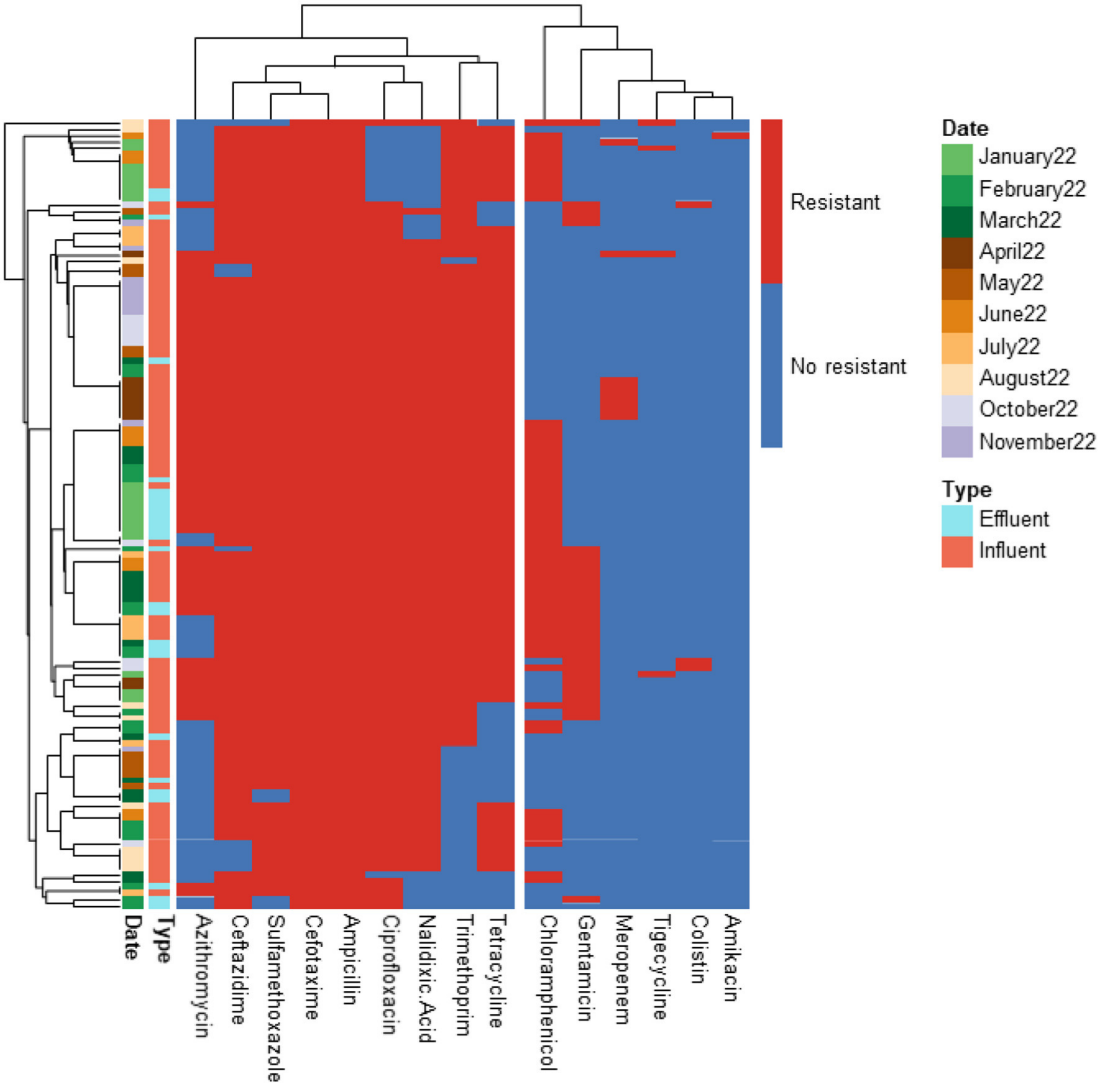
Heatmap displaying the antibiotic resistance profile of ESBL-producing *E. coli* isolates retrieved from PAA/UV Low WWTP. The isolates (*n* = 126) were classified as antibiotic resistant (in red) or susceptible (in blue) based on ECOFF values from EUCAST, which served as the threshold.

**Figure 4:**
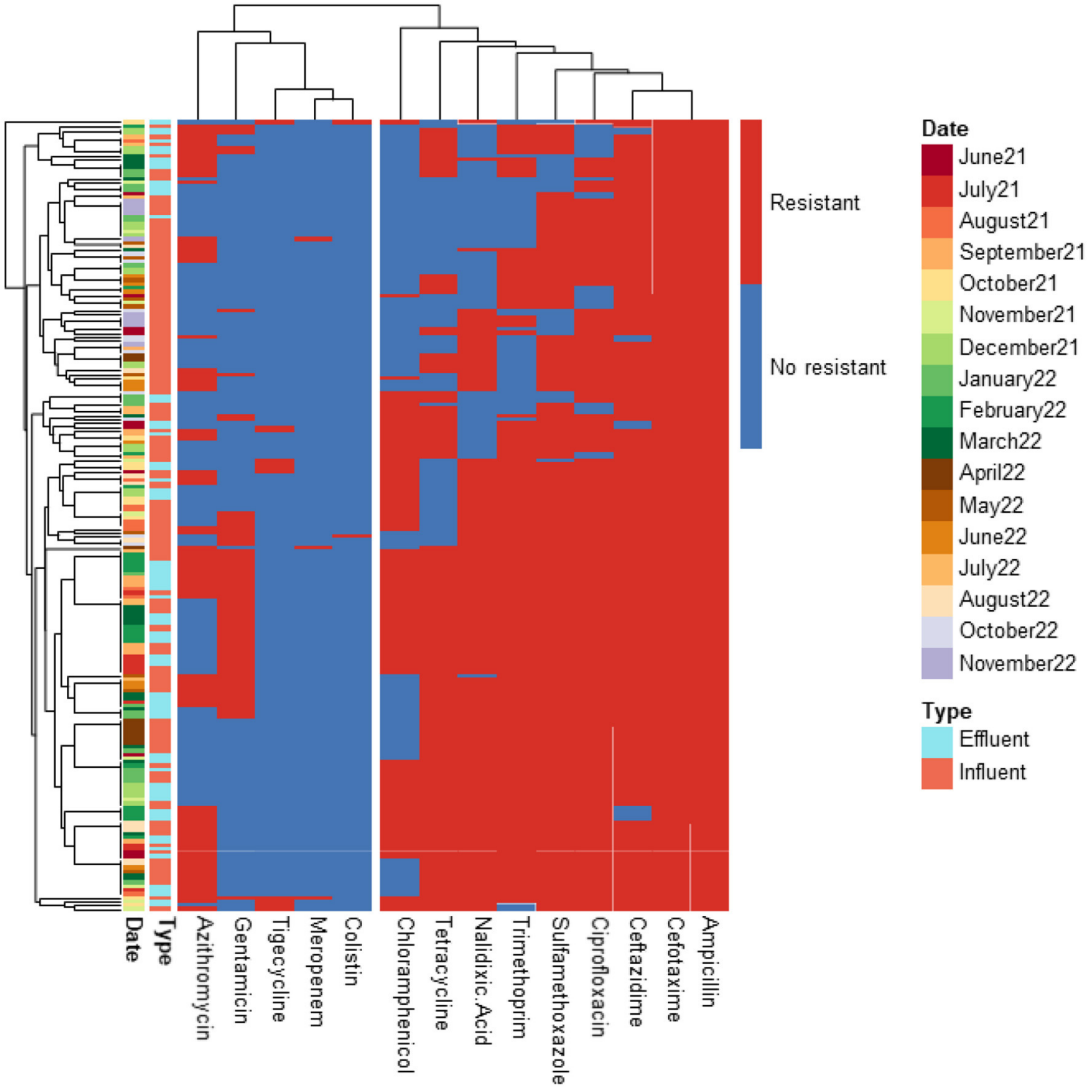
Heatmap displaying the antibiotic resistance profile of ESBL-producing *E. coli* isolates retrieved from UV High WWTP. The isolates (*n* = 210) were classified as antibiotic resistant (in red) or susceptible (in blue) based on ECOFF values from EUCAST, which served as the threshold.

**Figure 5:**
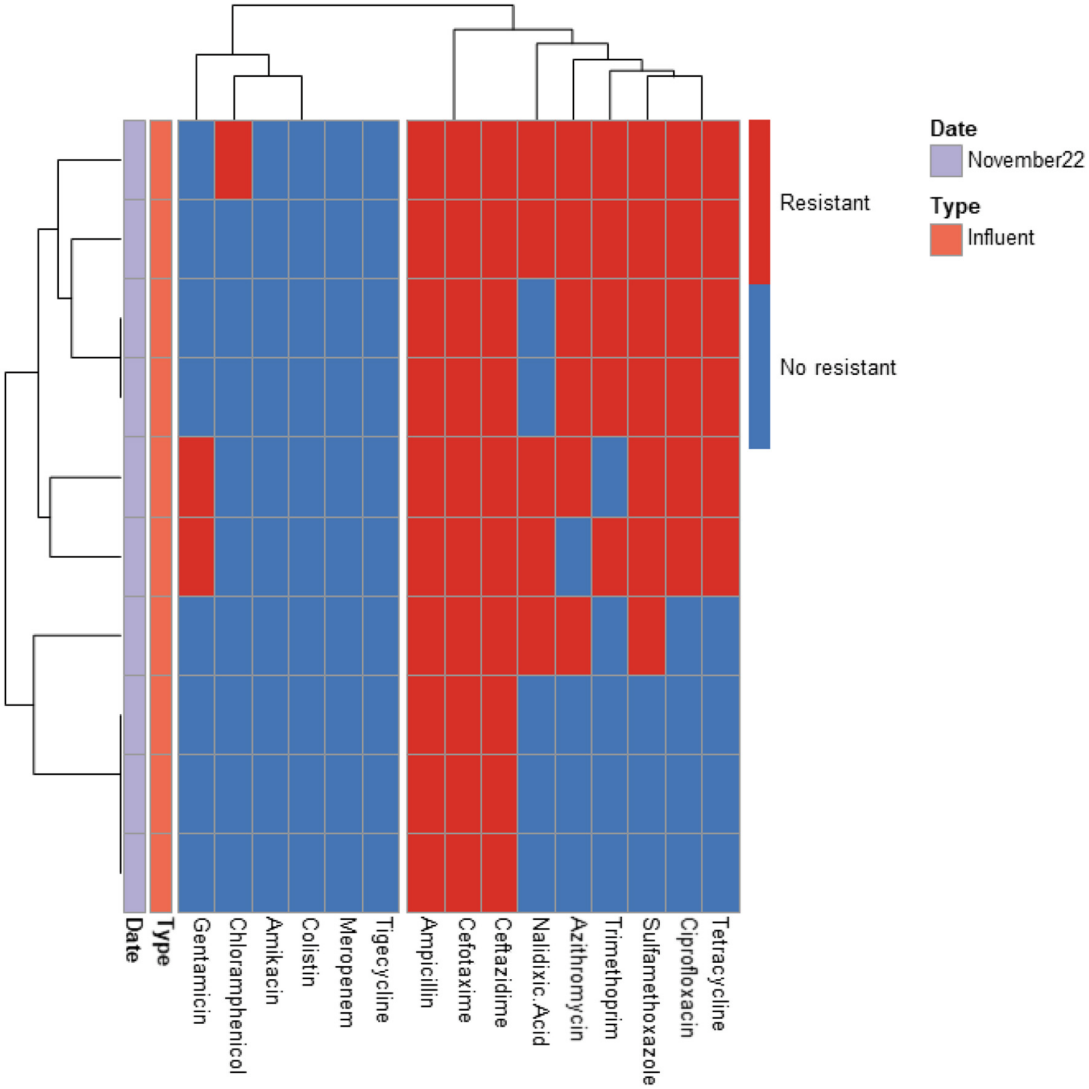
Heatmap displaying the antibiotic resistance profile of ESBL-producing *E. coli* isolates retrieved from UF WWTP. The isolates (*n* = 10) were classified as antibiotic resistant (in red) or susceptible (in blue) based on ECOFF values from EUCAST, which served as the threshold.

A total of 366 ESBL-producing *E. coli* isolates from influent and effluent samples of the four WWTPs were analyzed. The antibiotic resistance profiles for 210, 126, 20, and 10 isolates from PAA, PAA/UV Low, UV High, and UF WWTPs, respectively, are shown in Figures 2–5. All isolates exhibited resistance to multiple antibiotics, classifying them as MDR since they were resistant to at least three different antimicrobial classes.

The predominant resistance phenotypes included: β-lactams (cefotaxime, ceftazidime, ampicillin); Quinolones (ciprofloxacin, nalidixic acid); Tetracyclines (tetracycline); Sulfonamides (sulfamethoxazole); Dihydrofolate reductase inhibitors (trimethoprim). However, meropenem, tigecycline, colistin, and amikacin retained antibacterial activity against most isolates. A greater number of isolates (15 isolates) exhibited resistance to tigecycline compared to meropenem (12 isolates), colistin (5 isolates), and amikacin (2 isolates). The tigecycline-resistant isolates were found in the influent of the UV High and PAA/UV Low WWTPs, as well as in the effluent of the UV High WWTP. Most of the meropenem-resistant isolates were detected in the influent samples from the PAA/UV Low WWTP. Colistin-resistant isolates were identified in an effluent sample from the UV High WWTP and in influent samples from the UV High and PAA/UV Low WWTPs. The two isolates resistant to amikacin were originated from the influent samples from the UV High and PAA/UV Low WWTPs. Notably, two isolates from the influent of UV High and PAA/UV Low showed combined resistance to tigecycline and meropenem, while one isolate from an effluent sample from the UV High WWTP showed combined resistance to tigecycline and colistin. The presence of these resistant phenotypes in extended-spectrum β-lactamase (ESBL)-producing isolates, although not predominant, is noteworthy. Glycylcyclines (tigecycline), polymyxins (colistin), and carbapenems (meropenem) are considered last-resort antibiotics, primarily recommended for treating infections caused by ESBL-producing Gram-negative bacteria. Resistance to these antibiotics was not detected in isolates from PAA and UF WWTPs. However, it is important to note that the number of isolates analyzed from these WWTPs was considerably lower, which may have influenced the results. Similarly, Amador et al. (2015) reported a high prevalence of antibiotic resistance in *Enterobacteriaceae* from WWTPs, including resistance to β-lactam antibiotics, tetracycline, ciprofloxacin, and trimethoprim/sulfamethoxazole. Other studies have also characterized AMR profiles of *E. coli* isolates from WWTPs, frequently detecting resistance to sulfonamides, tetracyclines, and aminopenicillins, while resistance to quinolones was reported at lower rates (Ferreira da Silva et al., 2006; Łuczkiewicz et al., 2010).

Our findings suggest that ESBL-producing *E. coli* isolates recovered from influent and effluent samples commonly exhibit multidrug resistance phenotypes, including resistance to β-lactams, quinolones, tetracyclines, and sulfonamides. While descriptive data did not show major shifts in resistance profiles between sample types or sampling dates, the limited number of isolates from some WWTPs prevent definitive conclusions. The detection of isolates resistant to last-resort antibiotics, particularly in effluent samples from certain WWTPs, warrants further investigation to clarify whether wastewater treatment processes may contribute to the selection or persistence of specific resistance phenotypes

Nonetheless, the presence of MDR in effluent samples remains a public health concern, as reclaimed water is frequently reused for irrigation in agricultural fields. This highlights the need for continuous monitoring of AMR in WWTPs to mitigate potential risks associated with environmental and human exposure.

### 3.3 Monitoring of absolute ARG abundance in reclamation treatments at WWTPs

**Figure 6:**
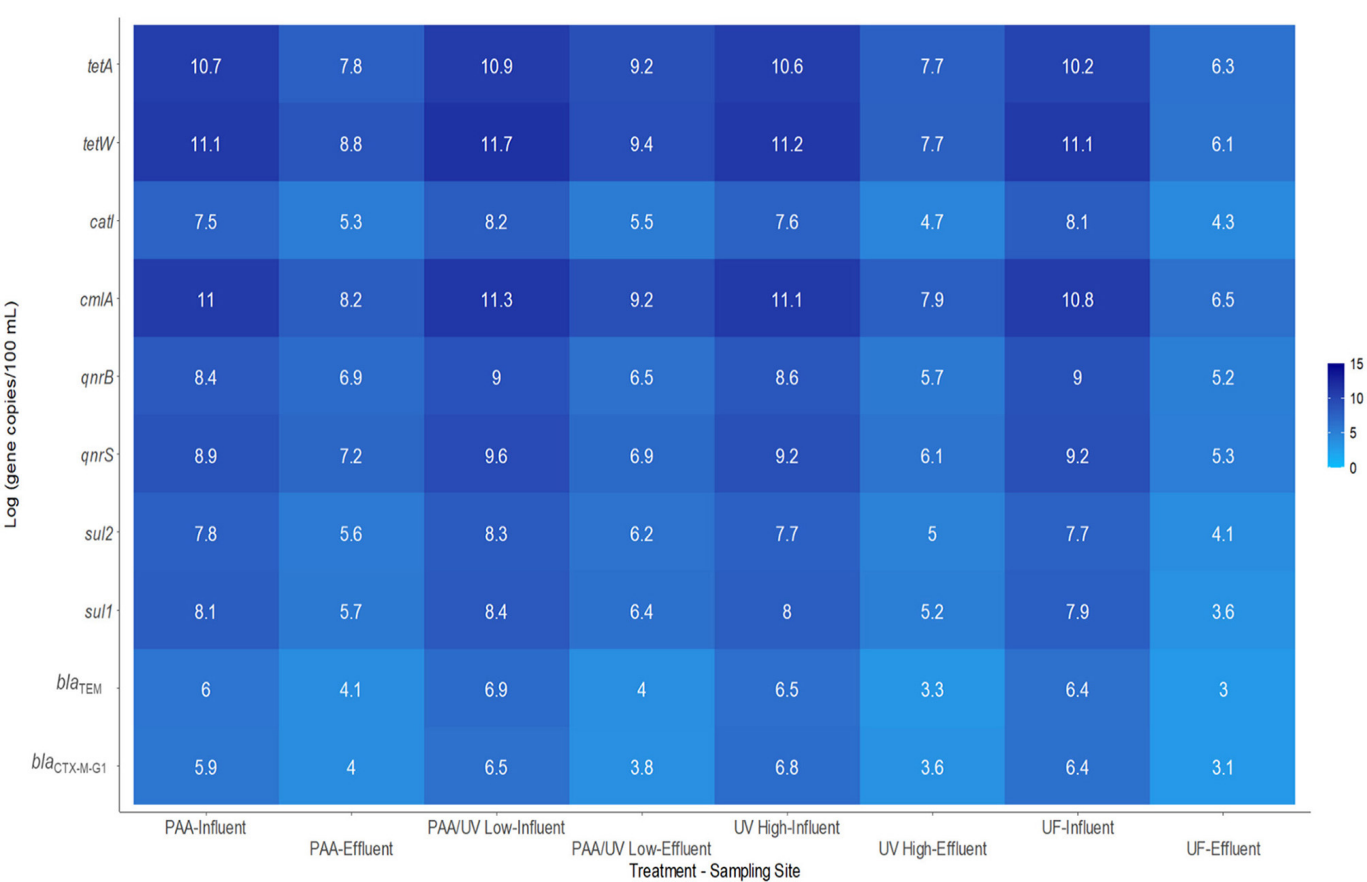
Heatmap representing the absolute levels of ARGs measured by qPCR in wastewater samples upon arrival at the WWTP studied and in its effluents after applying the tertiary treatment corresponding to each plant over an 18-month sampling period. Values are presented as Log (gene copies/100 mL).

In this study, the 16S rRNA gene and 10 ARGs were detected in all influent and effluent water samples from the four WWTPs (Table 3), except for the *sul1* gene in the effluent of the UV High WWTP and both *sul1* and *catl* genes in the effluent of the UF WWTP. These genes have been frequently reported in wastewater across various geographical regions, including Europe, America, Asia, and Africa (Oliveira et al., 2023; Pazda et al., 2019; Wang et al., 2020). In all WWTP influents, the most prevalent ARG was *tetW* (11.1–11.7 gc/100 mL), followed by *tetA* (10.2–10.9 gc/100 mL), *cmlA* (10.8–11.3 gc/100 mL), and *qnrS* (8.9–9.3 gc/100 mL; Figure 6).

**Table 3:** Prevalence of ARGs analyzed in the current study.

**WWTPs**	**Samples**	***bla*_CTX − M−G1_**	***bla*_TEM_**	***sul1***	***sul2***	***catI***	***cmlA***	***qnrB***	***qnrS***	***tetA***	***tetW***	***16S rRNA***
PAA	Influent	100 (18/18)	100 (18/18)	100 (18/18)	100 (18/18)	100 (18/18)	100 (18/18)	100 (18/18)	100 (18/18)	100 (18/18)	100 (18/18)	100 (18/18)
	Effluent	100 (18/18)	100 (18/18)	100 (18/18)	100 (18/18)	100 (18/18)	100 (18/18)	100 (18/18)	100 (18/18)	100 (18/18)	100 (18/18)	100 (18/18)
PAA/UV low	Influent	100 (18/18)	100 (18/18)	100 (18/18)	100 (18/18)	100 (18/18)	100 (18/18)	100 (18/18)	100 (18/18)	100 (18/18)	100 (18/18)	100 (18/18)
	Effluent	100 (18/18)	100 (18/18)	100 (18/18)	100 (18/18)	100 (18/18)	100 (18/18)	100 (18/18)	100 (18/18)	100 (18/18)	100 (18/18)	100 (18/18)
UV high	Influent	100 (18/18)	100 (18/18)	100 (18/18)	100 (18/18)	100 (18/18)	100 (18/18)	100 (18/18)	100 (18/18)	100 (18/18)	100 (18/18)	100 (18/18)
	Effluent	100 (18/18)	100 (18/18)	94 (17/18)	100 (18/18)	100 (18/18)	100 (18/18)	100 (18/18)	100 (18/18)	100 (18/18)	100 (18/18)	100 (18/18)
UF	Influent	100 (12/12)	100 (12/12)	100 (12/12)	100 (12/12)	100 (12/12)	100 (12/12)	100 (12/12)	100 (12/12)	100 (12/12)	100 (12/12)	100 (12/12)
	Effluent	100 (12/12)	100 (12/12)	42 (5/12)	100 (12/12)	83 (10/12)	100 (12/12)	100 (12/12)	100 (12/12)	100 (12/12)	100 (12/12)	100 (12/12)

The absolute abundance of ARGs in influent samples ranged as follows: (i) PAA: 5.9–11.1 gc/100 mL; (ii) PAA/UV Low: 6.5–11.7 gc/100 mL; (iii) UV High: 6.5–11.2 gc/100 mL; and (iv) UF: 6.4–11.1 gc/100 mL (Figure 6). On the other hand, effluent samples exhibited significantly lower ARG levels (*P* ≤ 0.05): (i) PAA: 4.0–8.3 gc/100 mL; (ii) PAA/UV Low: 3.8–9.4 gc/100 mL; (iii) UV High: 3.6–7.7 gc/100 mL; (iv) WWTP UF: 3.0–6.1 gc/100 mL (Figure 6). Consistent with influent samples, *tetW, tetA, cmlA*, and *qnrS* remained the most abundant genes in effluents (Figure 6). The high prevalence of tetracycline and quinolone resistance genes in wastewater and effluent is likely due to the widespread use of these broad-spectrum antibiotics. These findings align with previous studies demonstrating that tetracycline, quinolone, sulfonamide, and macrolide resistance genes are frequently detected in WWTP effluents (Pazda et al., 2019; Wang et al., 2020). While wastewater treatment reduced ARG abundance, significant levels were still detected in effluents, consistent with other reports (Luo et al., 2024; Munir et al., 2011; Pallares-Vega et al., 2019; Raza et al., 2022).

**Figure 7:**
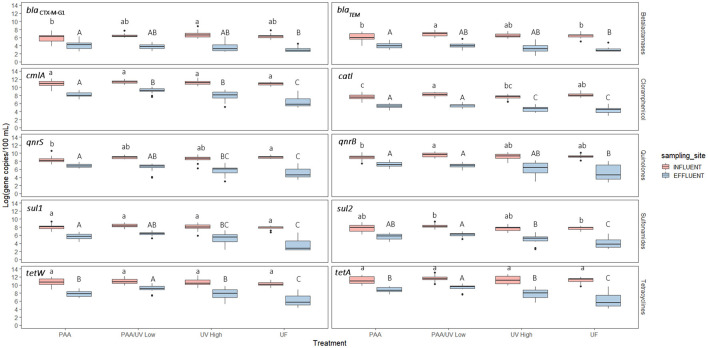
Absolute concentration of the 10 ARGs in the influent and effluent samples collected from each WWTPs. Box plots show median with the 25th and 75th percentile values of reduction. Box plots labeled with different lower-case letters indicate significant differences among influents at *P* < 0.05. Box plots labeled with different upper-case letters indicate significant differences among treatments at *P* < 0.05.

Significant differences were observed in ARG levels among the WWTPs (Figure 7). UF treatment achieved the most substantial reduction in ARGs, consistent with studies suggesting that membrane filtration is an effective approach for removing emerging contaminants (Guo et al., 2021; Zhang et al., 2023). This high effectiveness is likely due to the physical retention of bacterial cells and extracellular DNA fragments by the membrane pores, which are small enough to block ARG carriers. However, studies on ARG removal in reclaimed water systems using UF remain limited.

**Figure 8:**
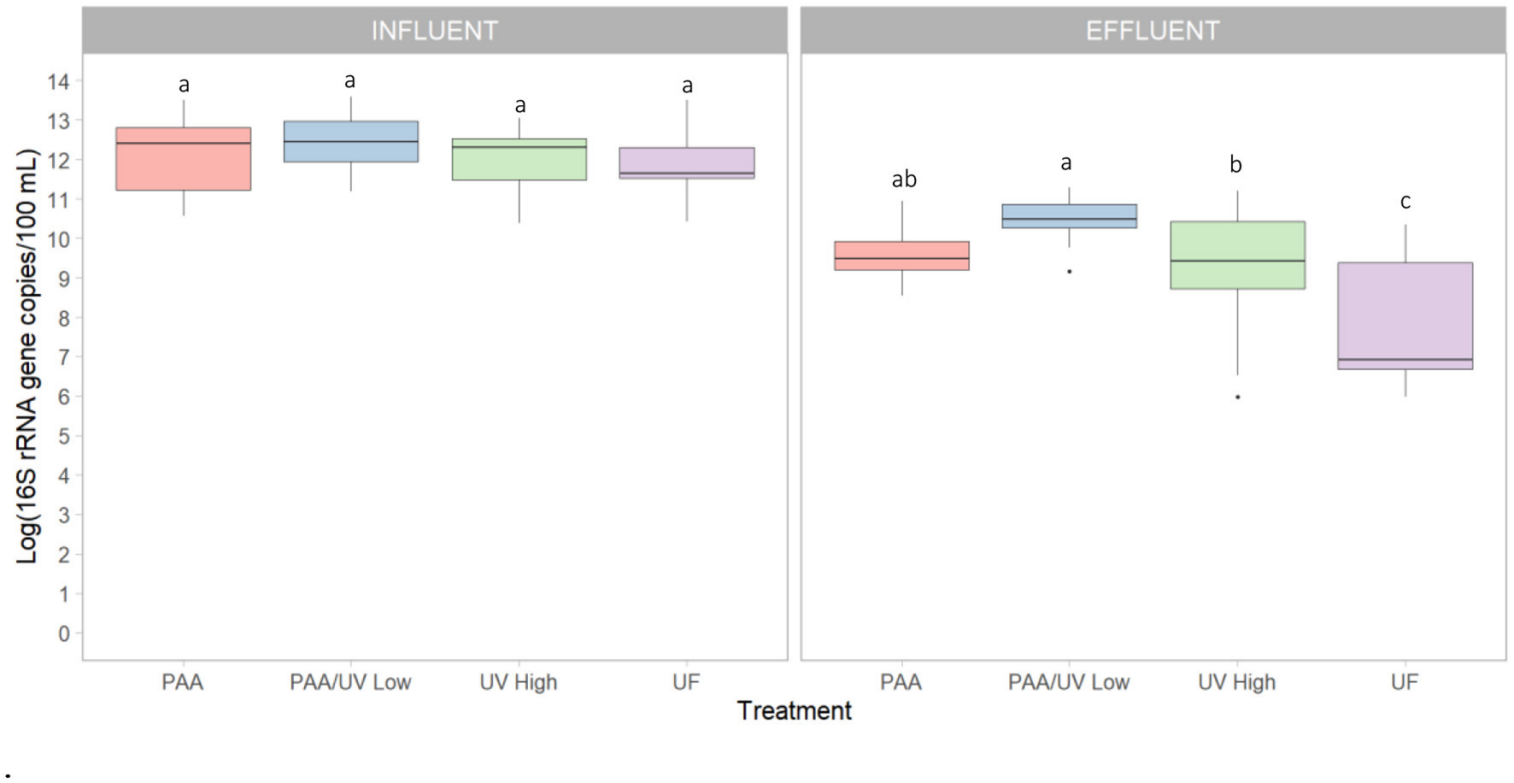
Absolute concentration of the *16S rRNA* gene in the influent and effluent samples collected from each WWTPs. Box plots show the median with the 25th and 75th percentile values of reduction. Box plots labeled with different letters indicate significant differences among treatments at *P* < 0.05.

Conversely, PAA/UV Low WWTP exhibited the highest absolute ARG levels in effluent, despite the fact that PAA/UV is often proposed as a promising disinfection strategy. However, a previous laboratory-scale study reported that PAA/UV Low treatment resulted in lower ARG levels than PAA or UV High alone (Ping et al., 2022). Several factors could explain these discrepancies. First, operational conditions such as variations in PAA dose, UV intensity, and contact time could have impacted the overall efficiency. For instance, if UV exposure or PAA concentration is insufficient or poorly synchronized, incomplete inactivation of microorganisms or ARG degradation may occur. In this particular case, the UV High doses used in that study (216 mJ/cm^2^**)** were lower than those applied in this study. All tertiary treatments reduced 16S rRNA gene levels, indicating a decrease in total bacterial load (Figure 8). UF showed the most significant reduction, suggesting its superior effectiveness in removing bacteria from wastewater. These results align with previous research evaluating 16S rRNA levels in WWTP influent and effluent (Pallares-Vega et al., 2019; Wang et al., 2021). The observed differences across WWTPs may therefore be influenced by a combination of treatment-specific parameters and site-specific factors, such as influent microbial load, composition of the wastewater, or presence of resistance-selection agents (e.g., residual antibiotics or heavy metals). These aspects highlight the complexity of ARG removal and suggest that further optimization and standardization of treatment parameters are essential to improve performance across different settings.

### 3.4 Monitoring of the relative abundance of ARGS in the water treatments applied at the WWTPs

**Figure 9:**
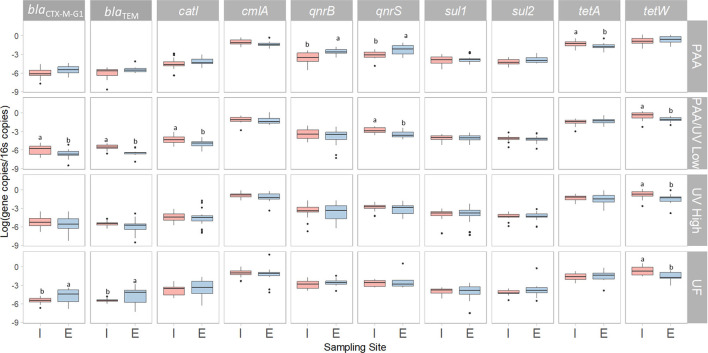
Relative abundance of ARGs in the influent (I) and effluent (E) samples collected from each WWTP. Box plots show the median with the 25th and 75th percentile values of reduction. Box plots labeled with different letters indicate significant differences between influent and effluent at *P* < 0.05.

To evaluate the impact of the four water treatments on ARG occurrence in WWTPs, the relative abundance of 10 ARGs was quantified as copies of ARGs per 16S rRNA gene copies using quantitative polymerase chain reaction (qPCR). The results showed that all four WWTPs either maintained or reduced the relative abundance of the analyzed ARGs, except for *bla*_CTX − M−G1_ and *bla*_TEM_ in the UF-treated effluent and *qnrB* and *qnrS* in the PAA-treated effluent (Figure 9). These findings align with previous studies, where some ARGs exhibited slight reductions or remained unchanged after wastewater treatment (Bengtson-Palme et al., 2016; Di Cesare et al., 2016; Laht et al., 2014). Similarly, McConnell et al. (2018) found no increase in ARG abundance following tertiary UV treatment at two WWTPs in Canada. Pallares-Vega et al. (2019) also reported no increase in *sul1, sul2, tetM, qnrS*, or *bla*_CTX − M_ relative abundance in 62 Belgian WWTPs. Conversely, other studies have reported increases in ARG levels following wastewater treatment, including Lee et al. (2017), Makowska et al. (2016), Marti et al. (2013), and Rodriguez-Mozaz et al. (2014). These conflicting results may stem from variations in microbial community composition post-treatment, the presence of AMR-selective agents in effluents, and differences in sampling design (Pallares-Vega et al., 2019).

### 3.5 Efficacy of tertiary treatments in WWTPs

**Figure 10:**
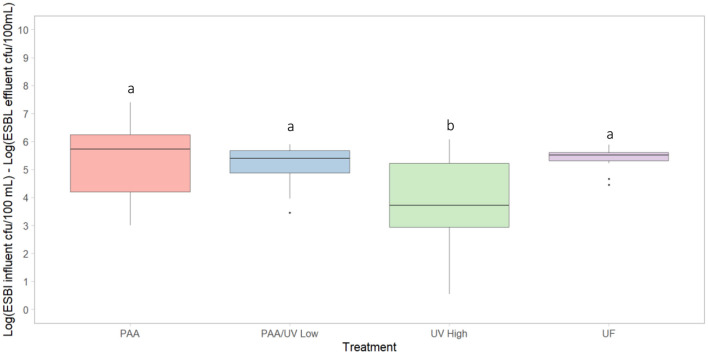
Box-plot representing the reduction of ESBL-producing *E. coli* after tertiary water treatments applied in each WWTP studied. The 0 log reduction (ESBL_I/ESBL_E) indicates no reduction of ARGs in the water. Box plots show the median with the 25th and 75th percentile values of reduction. Box plots labeled with different letters indicate significant differences among treatments at *P* < 0.05.

**Figure 11:**
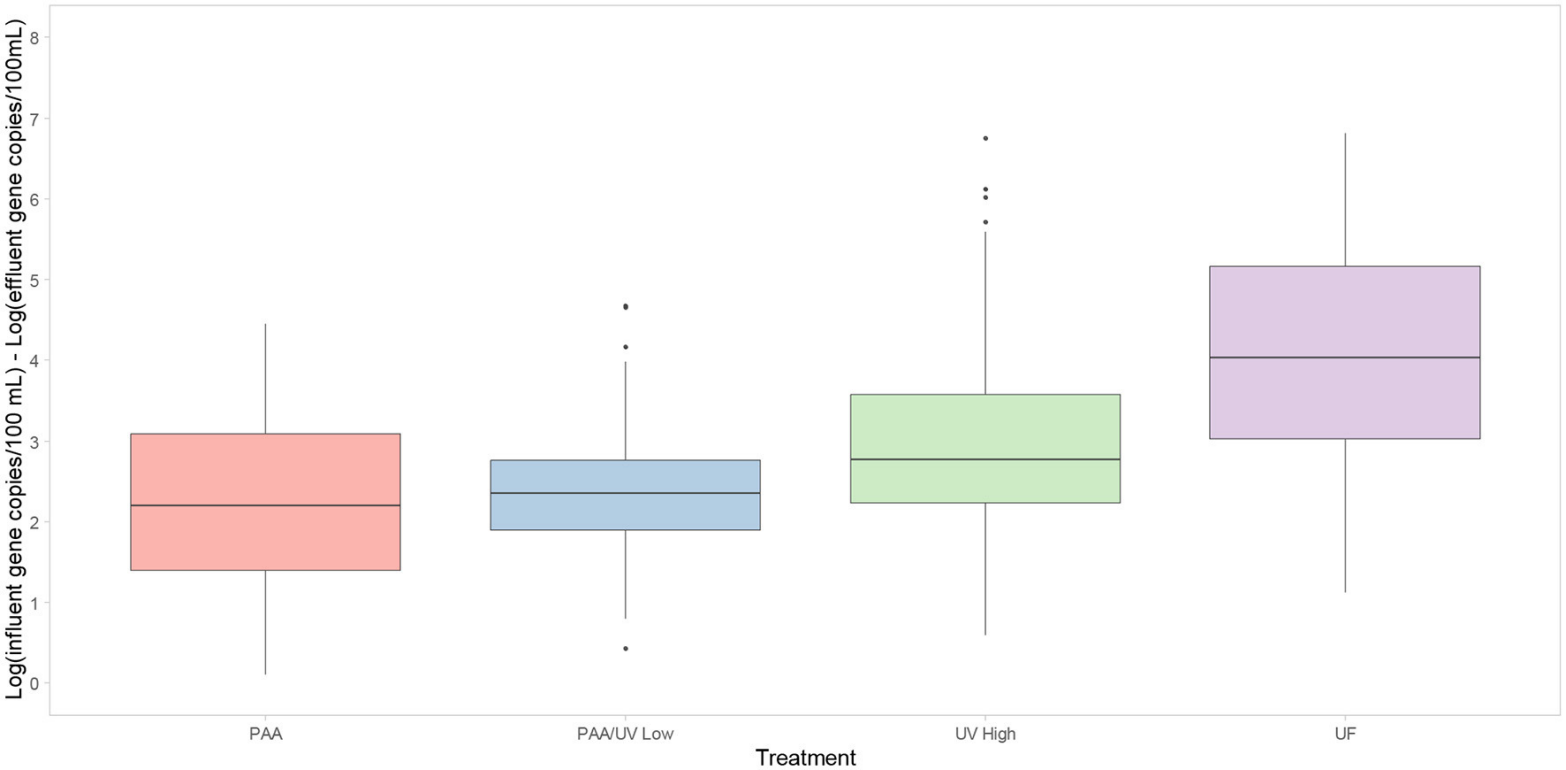
Box-plot representing the reduction of 10 ARGs evaluated after tertiary water treatments applied in each WWTP studied. The 0 log indicates no reduction of ARGs in the water. Box plots show median with the 25th and 75th percentile values of reduction. Box plots labeled with different letters indicate significant differences among treatments at *P* < 0.05.

Significant differences were observed between UV High and the other treatments (PAA, PAA/UV Low, and UF). The obtained results are consistent with studies evaluating the efficacy of UF, PAA/UV, and UV in removing ARBs (Balachandran et al., 2021; Chhetri et al., 2022; McKinney and Pruden, 2012; Michael et al., 2022; Oliveira et al., 2023; Raven et al., 2019). For instance, when UF is applied to activated sludge effluent achieved a 3–4 log reduction of enteric opportunistic pathogens and significant ARB removal (Michael et al., 2022). The application of a PAA dose of 4 mg/L for 7 min led to a 4-log reduction in multidrug-resistant *E. coli* (Balachandran et al., 2021). In fact, PAA treatment was effective in reducing ciprofloxacin-resistant bacteria in municipal and hospital wastewater (Chhetri et al., 2022). In the case of UV-C, this treatment alone or in combination with PAA or chlorine efficiently removed ESBL-producing *E. coli* from influent water (Oliveira et al., 2023). UV doses of 10–20 mJ/cm^2^ led to 4–5 log reductions in ARBs (McKinney and Pruden, 2012). In this study, UV High showed a lower reduction than the other treatments (Figure 10), suggesting lower efficacy in removing ESBL-producing *E. coli*. However, the median log reduction for UV High was ~3.5 logs, similar to the 3.2 log reduction observed in UV-treated effluents from a WWTP in southern England (Raven et al., 2019). Regarding ARG removal, the different WWTPs achieved an average reduction of 2.2–3.9 log/100 mL (Figure 11). These reductions are consistent with those observed in previous studies evaluating UV, PAA, UF, and PAA/UV combinations as tertiary treatments (Manoharan et al., 2022; Wang et al., 2020).

**Figure 12:**
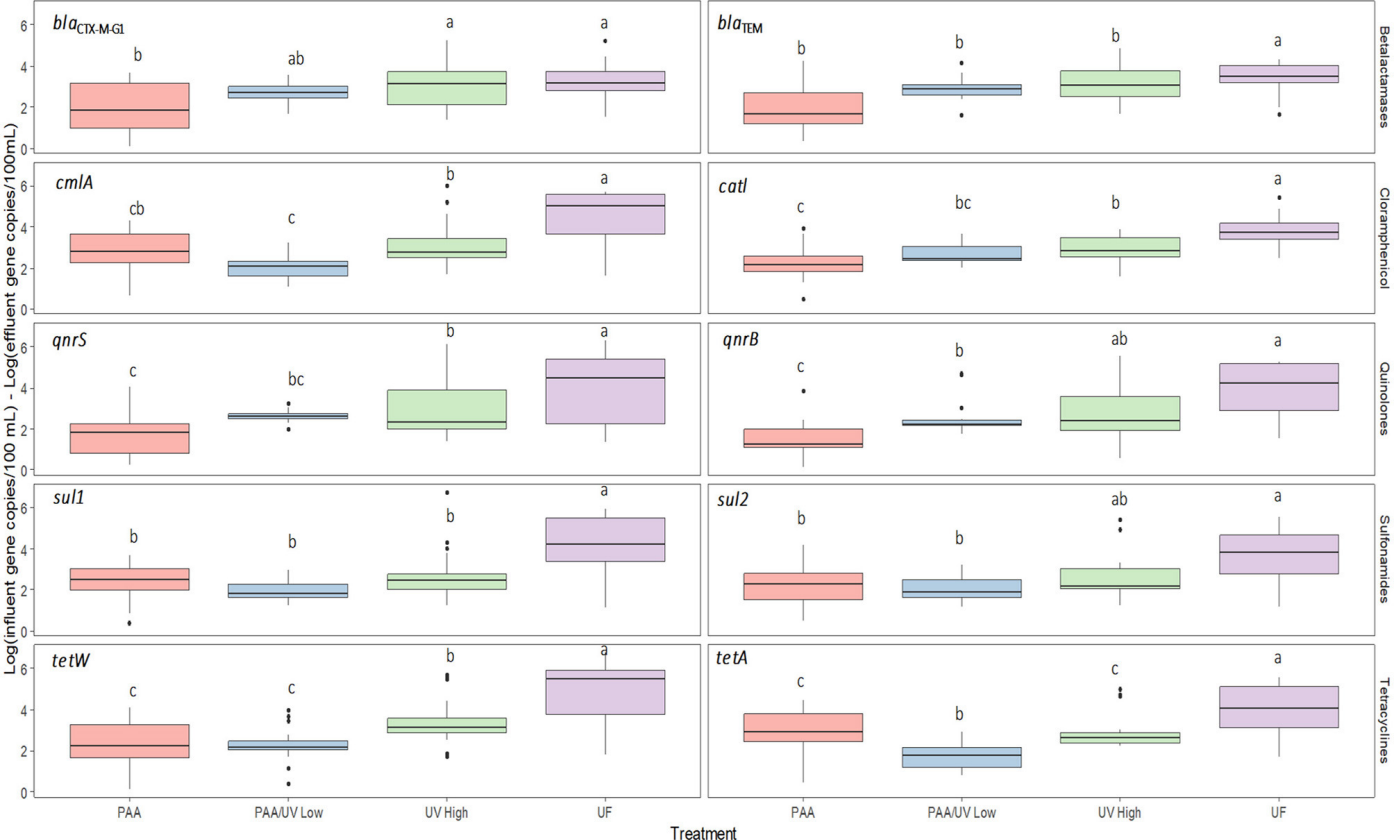
Reduction of each ARGS after tertiary water treatments applied in each WWTP studied. The 0 log indicates no reduction of ARGs in the water. Box plots show median with the 25th and 75th percentile values of reduction. Box plots labeled with different letters indicate significant differences among treatments at *P* < 0.05.

In the case of ARG, significant differences were observed among WWTPs. UF was the most effective treatment for reducing ARG concentrations. UV High followed UF in effectiveness, with better performance than PAA/UV Low and PAA alone. On the other hand, PAA/UV Low and PAA exhibited the lowest ARG removal efficiencies (Figure 12). These findings align with Liang et al. (2021), who reported that integrated membrane filtration reduced ARGs by nearly 3 logs (99.79%), from 3.02 × 108 to 6.45 × 105 cg/mL.

Several studies have demonstrated the ability of UV light to reduce ARGs in wastewater (Jia et al., 2021; Zhuang et al., 2014). However, other studies have reported minimal or no ARG reductions following UV treatment (Auerbach et al., 2007; Lee et al., 2017; Munir et al., 2011; Yang et al., 2019). These discrepancies are likely due to variations in UV dose. For instance, McConnell et al. (2018) found that WWTPs using 250 mJ/cm^2^ UV doses achieved greater ARG removal than those using lower doses (50 mJ/cm^2^). Studies suggest that combining PAA with low UV doses enhances ARG removal, but in this study, UV High alone was more effective than PAA/UV Low or PAA alone. This is likely due to higher UV doses applied in that WWTP. Ping et al. (2022) recently demonstrated that a high UV dose (108 mJ/cm^2^) and PAA (4 mg/L) effectively reduced ARGs, whereas lower UV doses (18 mJ/cm^2^) with the same PAA concentration were less effective. These findings highlight the role of tertiary treatments in mitigating ARG prevalence, emphasizing the importance of effective water treatments before discharge to reduce antibiotic resistance risks.

## 4 Conclusions

This study assesses the efficacy of different tertiary wastewater treatment technologies in reducing ESBL-producing *E. coli* and ARGs in reclaimed water intended for irrigating horticultural crops. The findings highlight the role of these treatments in limiting AMR dissemination and their implications for water reuse safety. Tertiary treatments significantly reduced ESBL-producing *E. coli* and ARGs, though complete elimination was not achieved. Ultrafiltration (UF) was the most effective, outperforming peracetic acid (PAA), PAA/UV Low, and UV High treatments. While absolute ARG abundance decreased across all WWTPs, relative abundance remained stable or only slightly reduced, with tetracycline (*tetW, tetA*), quinolone (*qnrS, qnrB*), and sulfonamide (*sul1, sul2*) resistance genes being the most persistent. UV efficacy was dose-dependent, with UV High performing better than PAA/UV Low and PAA alone, emphasizing the need to optimize UV intensity for ARG inactivation. Antibiotic resistance profiles revealed MDR *E. coli* in all WWTPs, primarily resistant to β-lactams, quinolones, tetracyclines, and sulfonamides. Additionally, resistance to last-resort antibiotics (tigecycline, colistin, meropenem) was detected at low frequencies, raising concerns about the potential dissemination of highly resistant bacterial strains through wastewater effluents. These findings underscore the need for improved wastewater treatment strategies and stricter monitoring to mitigate AMR risks in reclaimed water intended for agricultural use.

## Data Availability

The raw data supporting the conclusions of this article will be made available by the authors, without undue reservation.
